# Readability and Quality of Online Information on Osteoarthritis: An Objective Analysis With Historic Comparison

**DOI:** 10.2196/12855

**Published:** 2019-09-16

**Authors:** Kieran Edward Murray, Timothy Eanna Murray, Anna Caroline O'Rourke, Candice Low, Douglas James Veale

**Affiliations:** 1 St. Vincent’s University Hospital University College Dublin Dublin Ireland; 2 Beaumont Hospital Beaumont, Dublin Ireland

**Keywords:** osteoarthritis, arthritis, patient, readability, internet

## Abstract

**Background:**

Osteoarthritis (OA) is the most common cause of disability in people older than 65 years. Readability of online OA information has never been assessed. A 2003 study found the quality of online OA information to be poor.

**Objective:**

The aim of this study was to review the readability and quality of current online information regarding OA.

**Methods:**

The term osteoarthritis was searched across the three most popular English language search engines. The first 25 pages from each search engine were analyzed. Duplicate pages, websites featuring paid advertisements, inaccessible pages (behind a pay wall, not available for geographical reasons), and nontext pages were excluded. Readability was measured using Flesch Reading Ease Score, Flesch-Kincaid Grade Level, and Gunning-Fog Index. Website quality was scored using the Journal of the American Medical Association (JAMA) benchmark criteria and the DISCERN criteria. Presence or absence of the Health On the Net Foundation Code of Conduct (HONcode) certification, age of content, content producer, and author characteristics were noted.

**Results:**

A total of 37 unique websites were found suitable for analysis. Readability varied by assessment tool from 8th to 12th grade level. This compares with the recommended 7th to 8th grade level. Of the 37, 1 (2.7%) website met all 4 JAMA criteria. Mean DISCERN quality of information for OA websites was “fair,” compared with the “poor” grading of a 2003 study. HONcode-endorsed websites (43%, 16/37) were of a statistically significant higher quality.

**Conclusions:**

Readability of online health information for OA was either equal to or more difficult than the recommended level.

## Introduction

### Background

Osteoarthritis (OA) causes significant morbidity. It is the most common cause of disability in people older than 65 years and a major contributor to health care cost worldwide [1]. A wide variety of treatments exist, with varying evidence bases. These include physiotherapy, pharmacotherapy, surgery, and alternative therapies [2].

Patient education forms a crucial role in decision making, medication adherence, and disease self-management [3,4]. Traditionally, physicians represented the primary, or even sole, source of patient information. However, the patient-doctor relationship and the flow of information are changing. This is partially because of increasing internet access and an increase in the depth and breadth of online content. The number of patients searching online for health information and the number of health-related websites continues to grow, and there are now over 70,000 websites providing health information [5,6]. Most patients now use the internet for health care information [7].

Despite an increase in the availability and usage of online health information, the readability and quality of online health information is variable [8]. At present, a number of standardized validated tools are available to assess both the readability (Flesch Reading Ease Score, FRES; Flesch-Kincaid Grade Level, FKGL; and Gunning-Fog Index, GFI) and quality (*Journal of the American Medical Association*, JAMA benchmark criteria; DISCERN instrument) of online health care information.

Guidelines state health information aimed at the general public should be at a 7th to 8th grade reading level (United States) [9]. Previous studies of other medical conditions have found most online health care information to be above this reading level, rendering it inaccessible to many patients [10-12]. However, the readability of online OA content has never been assessed.

A 2003 study, using the standardized and validated DISCERN tool, graded online information concerning OA as poor [2]. Since then, there has been a marked increase in the number of websites from 40,912,332 in 2003 to 1,329,189,590 at present [13]. A recent study of the *knee osteoarthritis treatment* showed a significant difference in the quality of online information between countries speaking different languages [14].

### Objectives

Given the significant morbidity of OA, the lack of a previous study assessing readability, and the lack of any recent study (<10 years) assessing the quality of online information in relation to OA in general, the aims of this study were to assess both the readability and quality of current online OA information using 6 previously validated tools.

## Methods

### Internet Search Strategy

A total of 2 authors (KEM and TEM) familiar with the topic selected the 4 most appropriate commonly appearing disease-specific search terms for review: *osteoarthritis*, *osteoarthrosis*, *degenerative arthritis,* and *degenerative joint disease*. These were then searched across the 3 most popular UK search engines (Google, Bing, and Yahoo!), collectively representing over 98% of UK searches [15]. *Osteoarthritis* provided the most Web addresses, also known as URLs, and was thus chosen for analysis. As research has shown that patients are unlikely to search beyond 25 pages [16], the most-viewed (top ranking) 25 websites on each search engine were included [10,17].

Inclusion criteria were the first 25 pages from Google, Bing, or Yahoo! (n=75). Duplicate websites (n=31) and nonreadable websites (n=7) were excluded. The nonreadable websites were nontext pages (n=3), paywall-protected websites (n=2) and those inaccessible for geographic reasons (n=2). Of the 75 studied websites, 38 met exclusion criteria, and 37 were considered suitable for analysis. In cases of pagination of the webpage (where a single item was spread across sequential pages on the same website), the sequential pages were also assessed. All websites were reviewed in January 2018.

Website producer (the group responsible for hosting or publishing the website) was defined as health care provider, professional society, for-profit organization, or not-for-profit organization {including governmental organizations and nongovernmental organizations (NGOs)}. Where a website detailed dates for both content creation and last update, the most recent date was used when measuring website currency.

Website authors (and reviewers where specified) were categorized into doctor, other medical professional, nonspecified medical staff, nonmedical author, or not reported. Websites required explicit naming of authorship to comply with JAMA guidelines.

### Readability

Using an online analysis tool, the readability of each website was evaluated for 3 validated scores: FRES, FKGL, and GFI [18].

Published in 1948, the FRES calculates readability using the formula 206.835 − 1.015 (total words/total sentences) − 84.6 (total syllables/total words). This generates a difficulty grading from 0 to 100, with higher scores indicating easier readabliltiy [19]. FKGL was developed by the US Navy in 1975. It assesses readability with the following formula: 0.39 (total words/total sentences) + 11.8 (total syllables/total words) − 15.59 [20]. The GFI calculates readability with the formula 0.4 ([words/sentences] + 100 [complex words/words]). However, it also acknowledges a list of common words that are not considered complex, despite their syllable count. This forms an estimate as to the years of education required for readability [17,21].

### Quality

The quality of each selected website was evaluated using Health On the Net Foundation (HON) Code of Conduct (HONcode) classification, JAMA benchmark criteria, and DISCERN score, all of which have been previously validated [10,17].

Founded in 1995, HON is a nonprofit, nongovernmental organization, accredited to the Economic and Social Council of the United Nations. It was created to promote the spread of quality health information around the world [22]. HONcode is perhaps the best-known quality label for medical and health websites. It was created to help standardize the reliability of medical and health information available online [23,24]. HONcode certification identifies websites with quality and nonbiased health information designed for patients [23]. It examines numerous factors including disclosure of authors’ qualifications, attribution of sources, complementarity to the doctor-patient relationship, data protection, justifiability, transparency, and disclosure of funding sources and advertising. Over 8000 sites have been certified [25]. Each website was checked against the HONcode database.

Published in 1997, the JAMA benchmark criteria list 4 criteria that quality websites should fulfill. These are identification of authorship, identification of sources, specifying the date of creation/update, and disclosures (of ownership, advertising policy, sponsorship, and conflicts of interests) [26]. The presence or absence of each of these parameters was recorded. The content producer of the website was taken from the webpage itself or the Contact Us/About Us tab.

Published in 1999, DISCERN is an instrument created by British universities, the National Health Service, and the British Library [27]. It determines website quality and reliability by grading 16 items (concerning reliability, description of treatment choices, and overall rating) from 1 (inferior) to 5 (superior). The score ranges from 16 to 80, with a higher score indicating better-quality information [27]. Study grading was performed by a single author (KEM), with consensus joint scoring (TEM) in cases of uncertainty.

### Statistical Methods

Mean website age, JAMA benchmark criteria, and DISCERN score for each website were reviewed with 1-way analysis of variance (ANOVA). Analysis was performed by Prism 7 (GraphPad software). Significance was set at *P*<.05.

## Results

### Internet Search Strategy

*Osteoarthritis* was the most-searched term, with 33,960,000 results across Google, Bing, and Yahoo!. Thus, it was selected for further analysis. *Degenerative arthritis* (21,790,000), *degenerative joint disease* (7,720,000), and *osteoarthrosis* (1,139,000) yielded fewer results and were, therefore, disregarded.

**Figure 1:**
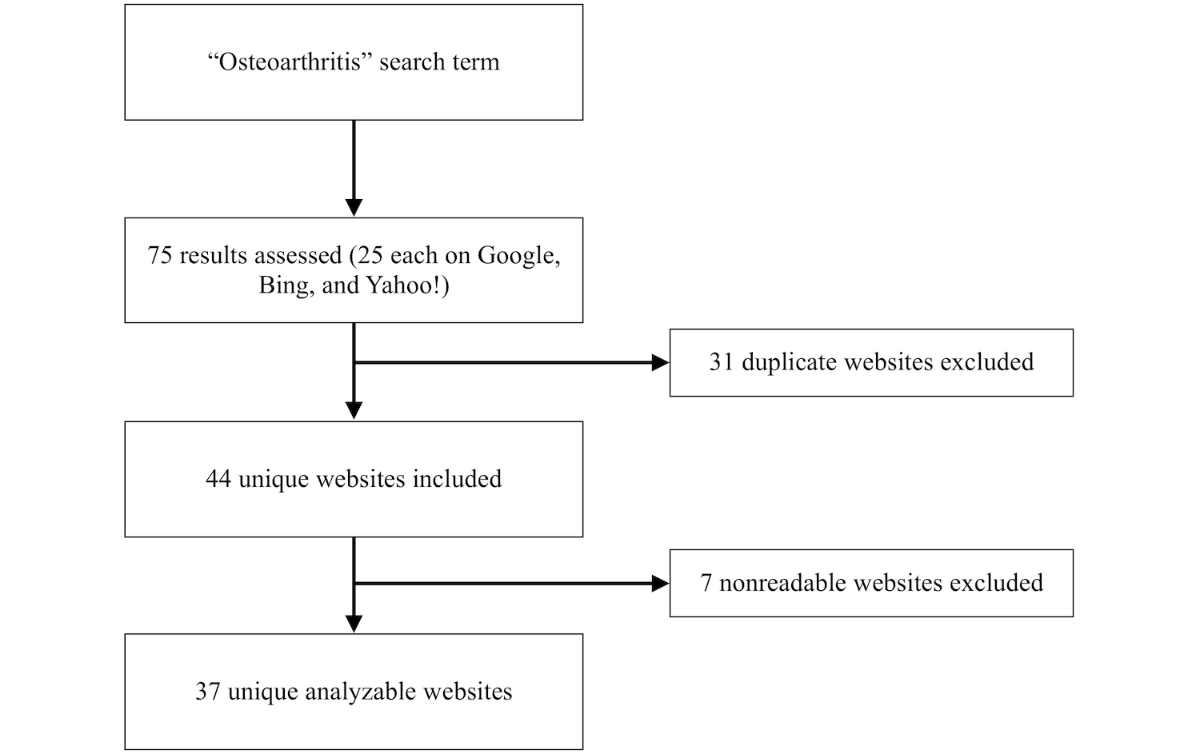
Internet search strategy (January 2018).

The internet search strategy is summarized in Figure 1. Of 75 articles, 38 met exclusion criteria. Of these, 31 were duplicate websites and 7 were nonreadable (nontext pages (n=3), paywall-protected websites (n=2), or inaccessible for geographic reasons (n=2). A total of 37 websites were considered suitable for analysis.

### Readability

The mean GFI of websites was 9.0, indicating a 9th grade reading level, mean FRES score was 51.4 (10th-12th grade reading ability), and mean FKGL score was 7.8 (8th grade). Readability scores by website content producer are shown in Tables 1-3.

There was no significant correlation between the type of organization publishing the website and readability, as measured by FRES (ANOVA *r*^2^=0.01; *P*=.93), FKGL (*r*^2^=0.01; *P*=.13), or GFI (*r*^2^=0.02; *P*=.88). Similarly, there was no significant correlation between the author type and website readability, as measured by FRES (ANOVA *r*^2^=0.18; *P*=.09), FKGL (*r*^2^=0.20; *P*=.06), or GFI (*r*^2^=0.14; *P*=.16).

**Table 1:** Quality and readability of online information on osteoarthritis.

Producer	Mean age (years)	HONcode^a^ certified, n (%)	Mean DISCERN score			
			Readability	Treatment choices	Quality	Total
All	1.4	16 (43)	23.1	16.6	2.6	42.3
Not-for-profit (governmental and NGOs; n=14)	0.9	3 (21)	24.1	17.2	2.6	43.9
Professional society (n=4)	1.3	0 (0)	22.6	16.3	2.5	41
For-profit organization (n=15)	1.5	12 (80)	23	16.9	2.6	42.5
Health care providers (n=4)	0.4	1 (25)	20.5	14	2.3	36.8

^a^HONcode: Health On the Net Foundation Code of Conduct.

**Table 2:** Quality and readability of online information on osteoarthritis.

Producer	Fulfill JAMA^a^ benchmark criteria, n (%)			
	Authorship	Attribution	Currency	Disclosure
All	11 (30)	9 (24)	22 (59)	9 (24)
Not-for-profit (governmental and NGOs; n=14)	0 (0)	3 (21)	7 (50)	3 (21)
Professional society (n=4)	1 (50)	2 (25)	3 (75)	1 (25)
For-profit organization (n=15)	8 (53)	4 (27)	11 (73)	5 (33)
Health care providers (n=4)	0 (0)	1 (25)	1 (25)	0 (0)

^a^JAMA: Journal of the American Medical Association.

**Table 3:** Quality and readability of online information on osteoarthritis.

Producer	Mean readability score		
	FRES^a^	FKGL^b^	GFI^c^
All	51.4	7.8	9.0
Not-for-profit (governmental and NGOs; n=14)	50.8	7.9	9.4
Professional society (n=4)	49.4	8.2	8.8
For-profit organization (n=15)	53.2	7.6	8.6
Health care providers (n=4)	48.5	8.2	9.2

^a^FRES: Flesch Reading Ease Score.

^b^FKGL: Flesch-Kincaid Grade Level.

^c^GFI: Gunning-Fog Index.

### Journal of the American Medical Association Benchmark Criteria

Each website was checked for compliance with JAMA benchmark criteria. Overall, 1 of 37 websites (3%) detailed author, attribution, currency, age, and disclosures. This was written by a doctor for a for-profit organization. For 23 websites (62%, 23/37) the author was not reported. Reported authors/reviewers were doctors (n=8), other health professionals (n=3), nonmedical author (n=2), and nonspecified medical staff (n=1). A total of 59% (22/37) of websites recorded the date of publication or update. The mean content age was 522 days (1.4 years).

**Figure 2:**
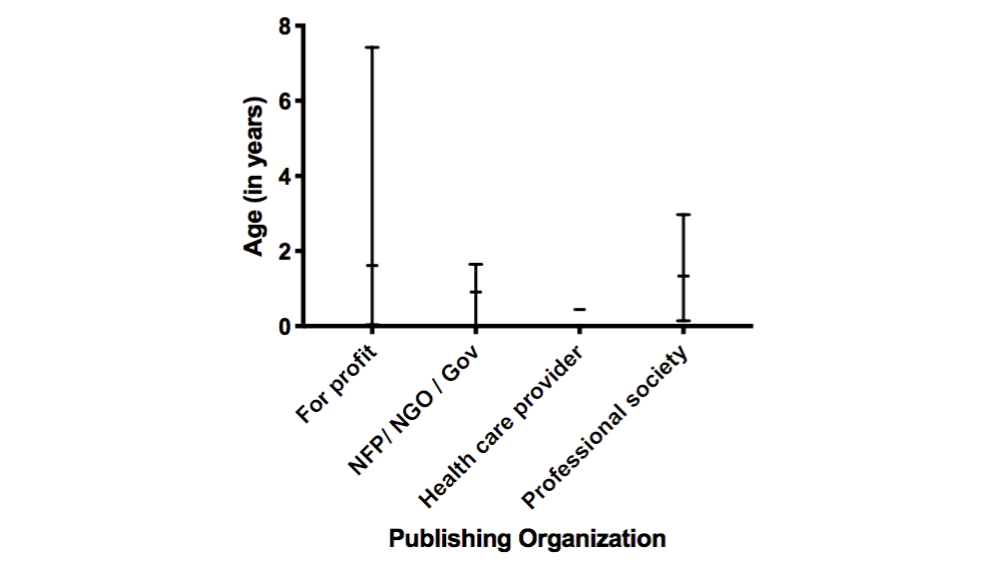
Website age by publishing organization type. Horizontal dash indicates mean and bar represents range. NFP: not for profit; NGO: nongovernmental organization.

The currency of the websites (time of last update or creation, whichever was most recent) did not significantly vary between publishing organization types (ANOVA *r*^2^=0.05; *P*=.79; Figure 2). There was no correlation between the search engine ranking of the website and JAMA score (*r*=−0.004; *P*=.97).

### DISCERN Score

The mean DISCERN scores for each of the 16 assessments of quality are shown in Multimedia Appendix 1. Overall, mean DISCERN score was 42.3. Thus, the quality of online health information for OA was “fair” [28], comparing favorably with the “poor” grading in 2003 [2]. The website with the highest DISCERN score (61) was *Mayo Clinic Patient Care and Health Information on Osteoarthritis* [29]. This website is also HONcode certified.

**Figure 3:**
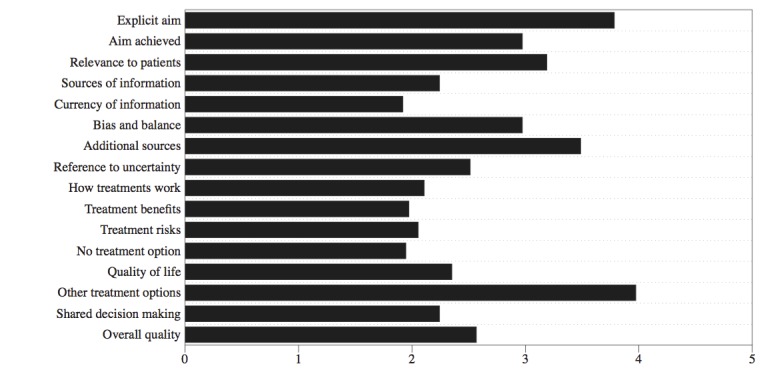
Relationship between website quality and author type. Box and whisker plot with horizontal line representing the median value, the box representing the interquartile range, and whiskers representing the range.

There was a significant difference in quality among author types (ANOVA *r*^2^=0.24; *P*=.03), with nondoctor health professional authors scoring the highest (mean 52.8 DISCERN score, SD 7.0) and nonmedical authors scoring the lowest (mean 24.0, SD 2.8; Figure 3). There was no correlation between the search engine ranking of the website and quality, as measured by either the DISCERN score (Spearman rank-order correlation *r*=0.05; *P*=.78). There was no significant difference in website quality (by DISCERN score) among publishing organization (ANOVA *r*^2^=0.14; *P*=.32).

### Health On the Net Foundation Code of Conduct Certification

Of 37 websites, 16 (43%) were HONcode certified. HONcode certification was significantly correlated with website quality as measured by DISCERN (unpaired 2-tailed *t* test; *P*=.001) and JAMA criteria (*P*=.02). HONcode certification was not, however, correlated with readability, as measured by FRES (*P*=.32), FKGL (*P*=.28), or GFI (*P*=.63) scores.

Rates of HONcode certification varied significantly among different publishing organization types (ANOVA *r*^2^=0.34; *P*=.002). This was highest among for-profit organizations (80%), with health care providers (25%), not-for-profit organizations (21%), and professional societies (0%) faring worse (Multimedia Appendix 2).

## Discussion

The mean readability of the websites differed somewhat by scoring technique. GFI indicated a 9th grade reading level. Mean FRES readability score was at the level of 10th to 12th grade reading ability, and FKGL score suggested an 8th grade reading ability. Thus, a large section of these websites exceeded the recommended 7th to 8th grade levels.

The quality of online OA health information (as per DISCERN score) has improved from “poor” in a 2003 study to “fair.” HONcode certification significantly correlated with website quality. There was a significant difference in quality between author types. Interestingly, doctors did not rank highest (although it is possible that they may have authored websites where the author was not reported). Nondoctor health professionals scored highest, followed by doctors and nonmedical authors.

Previous studies have shown that patients are much more likely to view pages with a higher search engine ranking [16]. We found no correlation between the search engine ranking of the website and quality as measured by either the DISCERN score or JAMA benchmark criteria. This indicates that a higher search engine ranking is not predictive of higher content quality. There was also no significant difference in website quality (by DISCERN score) among publishing organization.

HONcode certification was high relative to similar studies for other conditions [10,30]. This may reflect the high prevalence and widespread public interest in OA. HONcode certification significantly correlated with website quality (as measured by DISCERN and JAMA criteria), suggesting this certification may provide both clinicians and patients with a useful aid to predict high-quality online information.

There were several limitations to this study. Website search strategy was different from that of the comparative study performed in 2003 (reflecting the wider variety of website analytic tools now available and employed in this study). This reflects the much-changed nature of Web-searching behavior over the intervening 15 years. This difference in study modality must be considered when comparing study findings. Although the number of pages analyzed in this study was limited to those appearing high up in the search engine order, this reflects previously researched patient-research patterns, where pages beyond 25 are rarely viewed [16]. Analyzing additional pages may provide additional statistical certainty, but this would not reflect patient search patterns. A further limitation is the analysis of only 3 search engines, which could feasibly limit the applicability of the results. These combined search engines represent 98% of the target audience’s internet searches; however, it is unlikely that additional search engine inclusion in this study would add value [15].

JAMA benchmarks and DISCERN criteria use a methodical approach to assess quality. Despite this, all such grading introduces some degree of subjectivity. Ultimately, patients are the intended target audience for this study. In this study, website scoring was performed by doctors. Regardless, the DISCERN criteria was developed for use by either health professionals or the general public, and numerous peer-reviewed studies have demonstrated high interrater agreement [27,31,32].

The readability assessment tools are objective and precise for written text, owing to their computerized calculation. Other website components, however, such as videos and images, can also affect understanding, and these are not analyzed using the readability assessment tools. This limitation has been noted in previous similar studies [17,33].

Internet use is widespread. As health care providers, it is important to develop and direct patients toward readable, high-quality online health information. This study suggests health care professionals should direct patients to HONcode-certified websites written by health care professionals, as these websites were of significantly higher quality. The single highest quality source is noted to be a patient information website from the Mayo Clinic [29]. From a policy perspective, readability remains an important issue. Online health information for the general public released by US governmental websites should comply with the National Library of Medicine guidelines (7th-8th grade reading level) [9]. When creating websites to provide patient information on OA, authors may use the JAMA benchmark criteria and DISCERN criteria to ensure compliance with quality standards. Authors should consider testing readability levels to ensure the content is appropriately targeted at a 7th to 8th grade reading level. Finally, authors should consider HONcode certification to provide an external assessment of website quality.

## Abbreviations

ANOVA: analysis of variance
FKGL: Flesch-Kincaid Grade Level
FRES: Flesch Reading Ease Score
GFI: Gunning-Fog Index
HON: Health On the Net Foundation
HONcode: Health On the Net Foundation Code of Conduct
JAMA: Journal of the American Medical Association
OA: osteoarthritis

## Appendix

Multimedia Appendix 1 Mean DISCERN scores.

Multimedia Appendix 2 Health On the Net Foundation Code of Conduct (HONcode) certification by website publishing organization type. NFP: not for profit; NGO: nongovernmental organization.
