# Supplementary figures and images for: Readability and Quality of Online Information on Osteoarthritis: An Objective Analysis With Historic Comparison
Source: Interact J Med Res. 2019 Sep 16;8(3):e12855. doi: 10.2196/12855 (PMC6754692; doi:10.2196/12855)

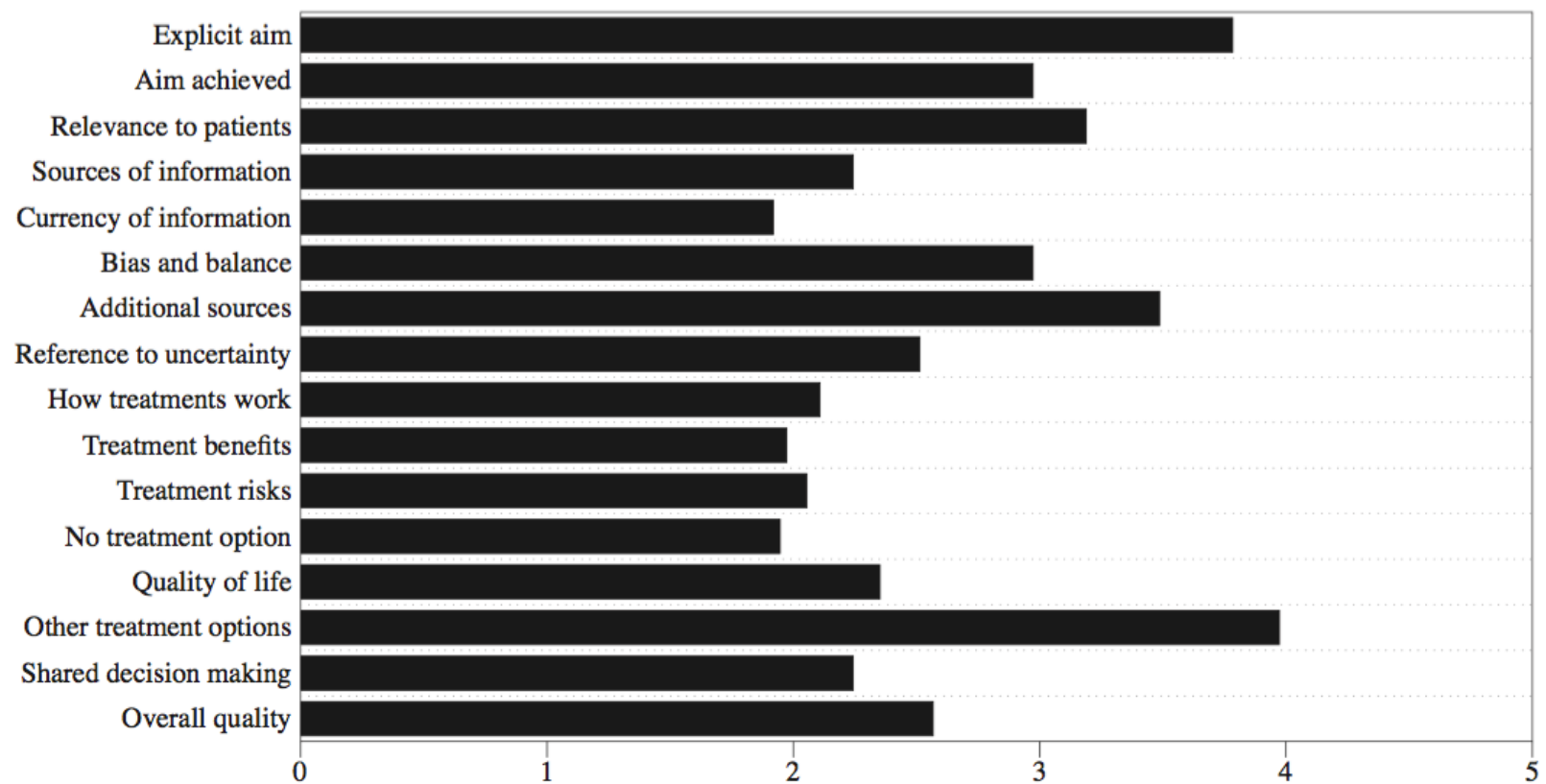

Supplement: Multimedia Appendix 1 [file ijmr_v8i3e12855_app1.pdf]

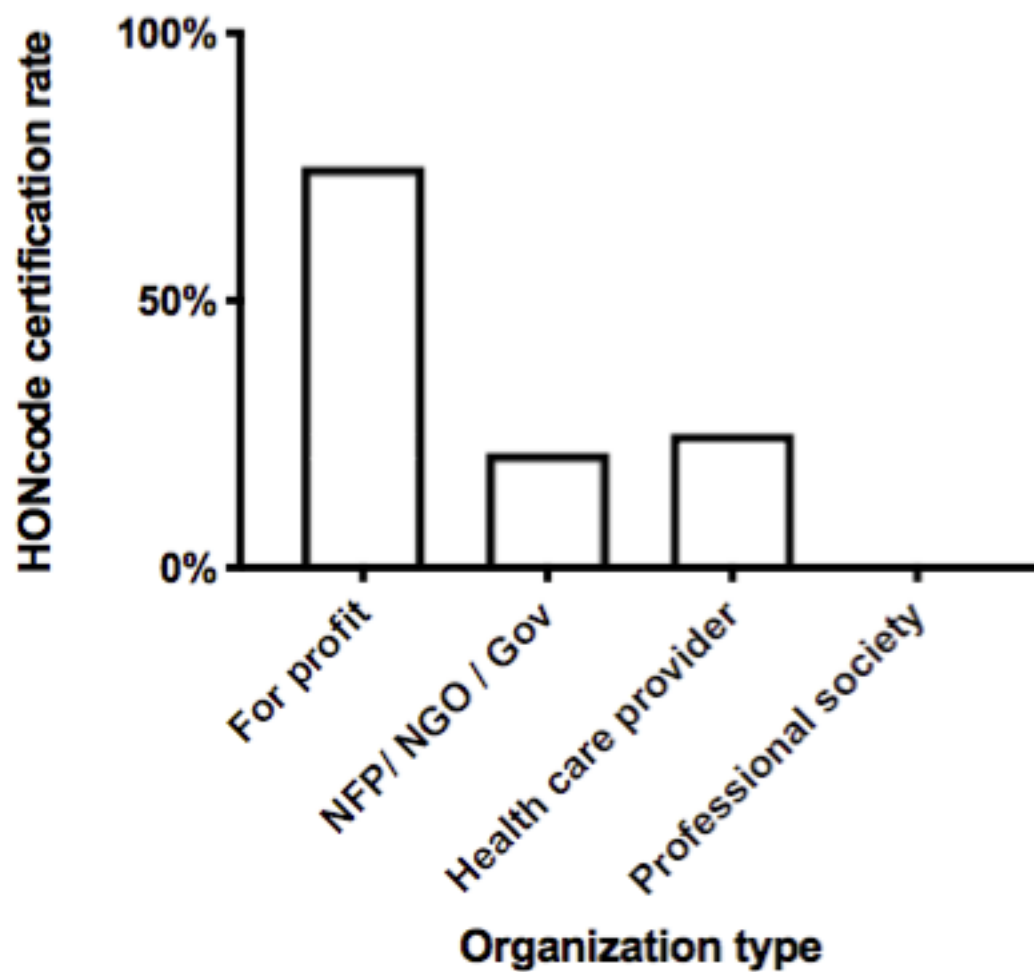

Supplement: Multimedia Appendix 2 [file ijmr_v8i3e12855_app2.pdf]
